# FOXM1 Transcriptionally Co-Upregulates Centrosome Amplification and Clustering Genes and Is a Biomarker for Poor Prognosis in Androgen Receptor-Low Triple-Negative Breast Cancer

**DOI:** 10.3390/cancers16183191

**Published:** 2024-09-18

**Authors:** Padmashree Rida, Sophia Baker, Adam Saidykhan, Isabelle Bown, Nikita Jinna

**Affiliations:** 1 Department of Science, Rowland Hall, Salt Lake City, UT 84102, USA; padmashreerida@rowlandhall.org (P.R.);; 2 City of Hope Comprehensive Cancer Center, Duarte, CA 91010, USA

**Keywords:** centrosome amplification, centrosome clustering, cell cycle, triple-negative breast cancer, quadruple-negative breast cancer, androgen receptor, KIFC1, HSET, FOXM1, racial disparity

## Abstract

**Simple Summary:**

Quadruple-negative breast cancer (QNBC; ER^−^, PR^−^, HER2^−^, and AR^−^) is a highly proliferative subtype of breast cancer that has no targeted treatment options. Chemotherapy, which is toxic to both malignant and healthy cells, is the mainstay treatment for this subpopulation. Centrosome amplification and clustering are cancer cell-specific traits that are upregulated in QNBC relative to other subtypes; thus, centrosome declustering drugs have been suggested to be a promising anticancer therapeutic strategy for this subgroup. However, the targeting of new centrosome biogenesis is neglected, rendering these drugs less effective. Herein, we propose targeting FOXM1, a master transcription regulator that drives the synchronous upregulation of centrosome amplification and clustering genes to circumvent this neglect. We discovered an overdrive of a FOXM1-mediated transcriptional signaling cascade in AR-low relative to AR-high triple-negative breast cancers (TNBCs). Hence, we assert that targeting FOXM1 may be a more efficacious anticancer strategy than centrosome declustering alone, suggesting FOXM1 could be a promising biomarker and actionable target in AR-low TNBC.

**Abstract:**

There are currently no approved targeted treatments for quadruple-negative breast cancer [QNBC; ER^−^/PR^−^/HER2^−^/androgen receptor (AR)^−^], a subtype of triple-negative breast cancer (TNBC). AR-low TNBC is more proliferative and clinically aggressive than AR-high TNBC. Centrosome amplification (CA), a cancer hallmark, is rampant in TNBC, where it induces spindle multipolarity-mediated cell death unless centrosome clustering pathways are co-upregulated to avert these sequelae. We recently showed that genes that confer CA and centrosome clustering are strongly overexpressed in AR-low TNBCs relative to AR-high TNBCs. However, the molecular mechanisms that index centrosome clustering to the levels of CA are undefined. We argue that FOXM1, a cell cycle-regulated oncogene, links the expression of genes that drive CA to the expression of genes that act at kinetochores and along microtubules to facilitate centrosome clustering. We provide compelling evidence that upregulation of the FOXM1-E2F1-ATAD2 oncogene triad in AR-low TNBC is accompanied by CA and the co-upregulation of centrosome clustering proteins such as KIFC1, AURKB, BIRC5, and CDCA8, conferring profound dysregulation of cell cycle controls. Targeting FOXM1 in AR-low TNBC may render cancer cells incapable of clustering their centrosomes and impair their ability to generate excess centrosomes. Hence, our review illuminates FOXM1 as a potential actionable target for AR-low TNBC.

## 1. Introduction

Breast cancer is the leading cancer diagnosis in women in the United States [1]. Based on the expression of hormone receptors and amplification of the human epidermal growth factor receptor 2 (Her2), breast cancer is divided into four molecular subtypes: Luminal A (ER^+^/PR^+^/HER2^−^), Luminal B (ER^+^/PR^+/−^/HER2^+^, or ER^+^/PR^+^/HER2^−^ with KI67 ≥ 14%), HER2-enriched (ER^−^/PR^−^/HER2^+^), and triple-negative breast cancer (ER^−^/PR^−^/HER2^−^, TNBC) [2]. BC subtypes that express ER and/or show Her2 amplification are effectively treated by therapies that target these receptors; as a result, TNBC, which is identified by the absence of these actionable targets, currently has no approved targeted therapies for use outside of clinical trials and is primarily managed with cytotoxic chemotherapy, radiation, and surgery [3,4]. In TNBC patients who experience a pathological complete response to neoadjuvant chemotherapy, the prognosis is fairly good. However, the remaining patients, who do not show a pathological complete response, have a high frequency of recurrence and metastasis within 5 years of diagnosis and, thus, a dismal prognosis [5]. While the incidence rates of breast cancer are similar among Black and White women (126.7 vs. 130.8 per 100,000), Black women experience a 40% higher mortality rate than White women. The fact that Black women are twice as likely to be diagnosed with TNBC than White women (38 vs. 19 per 100,000) may contribute to this stark racial disparity [6]. Additionally, TNBC patients are a highly heterogeneous group, and several classification systems have attempted to categorize TNBCs into distinct molecular subtypes based on gene expression profiles [7,8]. Spurred by the identification of androgen receptors (AR) as potential treatment targets in prostate cancer, clinicians and researchers have proposed the use of AR expression (as assessed by immunohistochemistry) as a criterion to stratify TNBC patients for AR-targeted therapies. Unfortunately, depending on the threshold used to define AR-positivity, ~65–88% of TNBC patients are AR-negative and are thus classified as having quadruple-negative breast cancer or QNBC, and AR-targeted treatments are unlikely to significantly benefit this patient subgroup [9]. QNBCs are an aggressive tumor subtype, as they have been identified to exhibit a basal-like molecular phenotype, including higher rates of TP53 mutations, to be highly proliferative, are diagnosed at a younger age, and are associated with a significantly shorter disease-free survival period, compared to TNBC [10,11,12,13,14]. QNBCs also show higher levels of chromosomal instability, centrosome amplification gene signatures, copy number alterations, and deregulation of miRNA expression [15]. Racial differences in QNBC biology have also been observed. African-American QNBC tumors show distinct gene expression profiles compared to White QNBC tumors; genes such as E2F1, NFKBIL2, CCL2, TGFB3, CEBPB, PDK1, IL12RB2, IL2RA, and SOS1 are differentially expressed, and immune checkpoint inhibitors PD-1, PD-L1, and CTLA-4 are overexpressed in Black compared to White QNBC tumors [13]. Once more, Black women are more likely to be diagnosed with QNBC, further reducing available treatment targets and contributing to racial disparities in breast cancer outcomes [10]. Hence, there is an urgent need for the identification of molecular drivers of aggressive QNBC tumor biology that may illuminate potential avenues for therapeutic intervention, which could aid in closing the racial gap in breast cancer outcomes.

## 2. Partners in Crime: Centrosome Amplification and Centrosome Clustering Collude to Drive Aggressive Breast Cancer

Malignant cells often contain an excessive number of centrosomes and/or centrosomes with an abnormally high volume, gained in a process known as centrosome amplification [16]. This phenomenon is so widespread that centrosome amplification is now recognized as a tumor hallmark. Centrosome amplification can occur as a result of cell—cell fusion, centrosome fragmentation, de novo centriole formation, or cytokinesis failure [17]. Overexpression of several genes is known to drive centrosome amplification; a gene expression-based signature called the CA20 signature, which computes the sum of the normalized (log2 median-centered) expression levels of the 20 centrosome structural genes and genes whose dysregulation induces CA (AURKA, CCNA2, CCND1, CCNE1, CDK1, CEP63, CEP152, E2F1, E2F2, LMO4, MDM2, MYCN, NDRG1, NEK2, PIN1, PLK1, PLK4, SASS6, STIL, TUBG1), was found to have a strong prognostic value in breast cancer [18]. In interphase in a normal cell, the centrosome duplicates once per cell cycle and enables the construction of a fusiform bipolar spindle during mitosis. Thus, possessing only one centrosome before the S phase and two after the S phase is critical to the proper division of the parent cell into two and the faithful partitioning of genetic material among daughters [18]. In theory, cells that bear additional centrosomes are at risk for multipolar divisions that can lead to catastrophic high-grade aneuploidy and almost-certain cell death [19]. However, instead of being hobbled by the presence of extra centrosomes, cancer cells deploy a crafty mechanism called centrosome clustering to corral the excess centrosomes into two polar groups and construct a “pseudo-bipolar” mitotic spindle. It turns out that cells endowed with supernumerary centrosomes go through a transient intermediate multipolar spindle stage when there are several merotelic attachments (i.e., capture of an individual kinetochore by microtubules emanating from two or more centrosomes) before centrosome clustering pathways engage and gather multiple centrosomes into two polar clusters [20,21]. These merotelic attachments are responsible for chromosomal instability and low-grade aneuploidy that results from the presence of excess centrosomes in cancer cells [22]. Centrosome clustering thus offers multiple benefits to cancer cells—it ensures survival and continued proliferation of cells harboring a surfeit of centrosomes, the persistence of amplified centrosomes in progeny cells, and the dogged maintenance of a low level of “tolerable” aneuploidy that fuels chromosomal instability, begets intratumoral karyotypic heterogeneity, fosters therapeutic resistance, and eventually promotes disease progression [18,19,23,24].

Effective centrosome clustering requires the collaboration of a large crew of proteins, many of which normally serve other essential roles in mitosis; this observation suggested that pre-existing cellular pathways were being hijacked by cancer cells to aid in managing the extra centrosomal load and to reap the collateral benefits that ensue from the “survivable” aneuploidy that centrosome amplification incites [18,19,23]. It is noteworthy that studies that compellingly showed that centrosome amplification is an early event in neoplastic transformation, simultaneously implied that centrosome clustering was also an early event in tumorigenesis because centrosome amplification is only survivable if centrosome clustering kicks in right away or if all additional centrosomes are inactivated; there are no known examples of the latter happening [25,26]. Additionally, while it is well recognized that the coaction of centrosome amplification and centrosome clustering mechanisms drives poor prognosis, little is known about whether connections exist between the two pathways that innately couple their expression in all cells and how cancer cells manage their co-upregulation. Regardless, throughout this process, cancer cells with excess centrosomes use the mitotic cell spindle assembly checkpoint (SAC) to their advantage. The SAC normally forestalls anaphase entry until all faulty attachments have been corrected and all chromosomes have bioriented on the mitotic spindle; the actions of the SAC thus ensure high fidelity chromosome segregation in all cells and provide time for centrosome clustering in cancerous cells [27].

## 3. Connecting the Dots: The Chromosome Passenger Complex (CPC) and KIFC1 Are Key Drivers of Centrosome Clustering and Transcriptional Targets of FOXM1

Many microtubule-binding proteins play vital roles in centrosome clustering. The most studied microtubule-binding protein, the minus-end directed kinesin-14 motor protein, KIFC1 (also known as Human Spleen, Embryo, and Testes motor protein or HSET), drives centrosome clustering in cancer cells by localizing between spindle microtubules, crosslinking, and sliding antiparallel microtubules and bundling them near the spindle poles to induce centrosome coalescence [28,29]. KIFC1 is highly upregulated in TNBC and imparts aggressive phenotypes to TNBCs, such as enhanced cell cycle kinetics, resistance to apoptosis, centrosome clustering ability, and taxane resistance [30,31,32]. KIFC1 inhibition impairs the proliferation and migration of African-American TNBC cells to a greater extent than that of European-American TNBC cells [32]. KIFC1 overexpression is associated with a survival rate of less than 5 years, which suggests that KIFC1 upregulation drives aggressive TNBC [33]. TNBC cells can be selectively eliminated by pharmacological inhibition of KIFC1 function without any apparent detrimental effects on non-malignant cells [30,31]. Furthermore, we recently discovered that the expression of KIFC1 and genes that drive CA is co-elevated in AR-low and AR-basal-like tumors relative to AR-high and AR-non-basal-like TNBC tumors [34]. This difference was most pronounced in TNBCs among African Americans. We also discovered that KIFC1 gene expression confers poorer survival among AR-low compared to AR-high TNBC patients [34]. Thus, KIFC1 is a promising prognostic biomarker and therapeutic target for TNBC, particularly for AR-low and African-American subpopulations.

However, KIFC1’s actions in isolation cannot guarantee both optimal centrosome clustering and a low and beneficial level of whole chromosome missegregation; KIFC1-mediated centrosome clustering would be subpar and entail a risk of high-grade aneuploidy without another mechanism regulating tension near the kinetochore [18]. In a study highlighting the potential mechanisms that suppress multipolar divisions in cancer cells with extra centrosomes, Kwon et. al., 2008 compiled a list of genes required for centrosome clustering [19]. In our effort to uncover links between CA and centrosome clustering pathways, we initially focused on the 31 shortlisted genes in Kwon et al., 2008, that had human homologs and were ranked as having a strong hit strength according to the’ siRNA screen. Because we were interested in the mechanisms that drive the aggressive tumor biology of AR-low TNBC, we further narrowed the scope of our research by identifying shortlisted centrosome clustering genes that were highly overexpressed in TNBC and whose expression was negatively correlated with the expression of AR in TNBC. The data from our analyses, presented in the sections below, illuminated the chromosomal passenger complex (CPC)—composed of Aurora B kinase, Inner Centromere Protein (INCENP), Survivin (BIRC5), and Borealin (CDCA8/Dasra)—as a key regulator of kinetochore-microtubule interactions, and an essential driver of both centrosome clustering and poor disease outcomes in AR-low TNBC [19,23].

During prometaphase, highly dynamic spindle microtubules capture the kinetochores they encounter as their plus ends explore the 3D space within the cell. If these kinetochore-microtubule attachments are low-tension ones (i.e., when the attachments produce a level of tension that is below the tension required to satisfy the SAC sensors), the SAC gets activated and provides time for these low-tension attachments to be fixed. In general, SAC sensors detect merotelic attachments rather poorly; as a result, merotelic attachments can persist. In prometaphase, cortical dynein molecules accumulate at opposite cellular poles, where they also capture astral microtubules; the subsequent minus-end directed movement of dynein exerts a force that pulls centrosomes toward two cellular poles. This complex multifocal tug-of-war that results from the motor activities of KIFC1 and dynein, the activity of the CPC at the kinetochore, and a host of other centrosome clustering molecules results in chromosomes lining up along the cell’s equator, as some centrosomes are pulled toward one spindle pole, and the remaining are pulled toward the other spindle pole [18]. Once all kinetochores experience the requisite tension or stretch, the SAC is satisfied, and anaphase can ensue. In the absence of CPC-dependent kinetochore-generated tension, the pulling forces produced by cortical dynein remain unopposed, centrosomes are pulled radially outward toward the cortex, and chromosomes are unable to congress to the cell’s equator efficiently. If KIFC1 is present and functional, it contributes to its pole-focusing activity; however, this alone is not sufficient to ensure robust coalescence of centrosomes at the two poles [18,23]. Leber et al., 2010 have also demonstrated unequivocally that in the absence of adequate spindle tension regulated by the CPC, centrosome clustering is ineffective, and spindle multipolarity and apoptosis result despite the actions of KIFC1 [23]. Thus, CPC-dependent kinetochore tension is a critical and hitherto under-appreciated force that must act in concert with the sliding and pole-focusing activities of KIFC1 closer to centrosomes to cluster excess centrosomes. It is, therefore, conceivable that effective clustering of centrosomes would require synchronous co-upregulation of KIFC1 as well as CPC genes.

Studies have shown that the localized enrichment of CPC components is a prerequisite for many of the complex’s functions—the CPC first localizes to inner centromeres and then associates with the macromolecular complex of the kinetochore. Finally, the CPC is associated with the midbody during cytokinesis [35]. The inner centromere is the region where sister chromatid cohesion is preserved until anaphase onset; the chromatin in this region has a specialized organization that enables sister chromatids to withstand the strong pulling forces that arise at the kinetochores as chromosomes biorient. The inner centromere is also a platform from which mitotic signals emanate. The CPC is believed to contribute in multiple ways to the production of the unique chromatin properties of the inner centromere [35]. The kinetochore, which connects chromosomal DNA to spindle microtubules, plays a critical role in generating the forces needed to correctly align chromosomes along the cell’s equator and segregate sister chromatids during anaphase. Trivedi et al., 2019 postulate that the liquid-liquid phase separation of the CPC, a process nucleated by Borealin, creates inner centromeric coacervates within which CPC complexes are concentrated and which are central to CPC’s functions in resisting the pulling forces of the spindle, and maintaining sister chromatid cohesion [36]. CPC components also play pivotal roles in the correction of faulty connections between chromosomes and spindle microtubules and in stabilizing correct kinetochore-spindle microtubule attachments [36,37].

In cancer cells, studies suggest that the CPC components are coordinately overexpressed as part of a cell cycle-regulated transcriptional program whose induction is strongly correlated with the expression of the Forkhead transcription factor FoxM1 that binds the promoters of these genes to drive their transactivation [38]. The importance of this synchronized upregulation is underscored by the idea that an uncoordinated increased expression of individual CPC proteins could lead to subunit imbalances and create dominant-negative effects that could adversely impact both the fidelity of chromosome segregation and the efficient clustering of amplified chromosomes. The same study also identified KIFC1 as a transcriptional target of FOXM1, suggesting that FOXM1 may be responsible for the synchronous expression of centrosome clustering genes that operate at kinetochores (e.g., CPC components) as well as those that function closer to spindle poles (e.g., KIFC1).

## 4. The Accomplices: CPC Components AURKB, BIRC5 and CDCA8 Play Distinct Roles in Promoting Mitotic Progression and Genomic Instability in Cancer Cells

AURKB is a member of an evolutionarily conserved family of Ser/Thr kinases and was first discovered in *Drosophila melanogaster* [39]. AURKB phosphorylates and regulates an astonishingly large array of substrates [40,41,42] and plays multiple crucial roles in prometaphase and metaphase, helping to create bipolar spindle attachments for sister chromatids. AURKB’s function, in conjunction with the activities of the other three CPC components, is also critical in anaphase, as the successful separation of sister chromatids during this phase is dependent on the dynamic regulation of kinetochore-microtubule attachment. The kinetochore complex grips rope-like spindle microtubules more strongly when there is greater tension, which ensures that kinetochores remain connected to microtubules that pull on and separate sister chromatids [43]. If the tension at the kinetochore is abnormally low, AURKB phosphorylates serine residues within NDC80, a protein that is essential for the attachment of kinetochores to spindle microtubules [44]. The phosphorylation of NDC80 causes the destabilization of microtubules that are improperly attached, creating another opportunity for proper/strong microtubule attachment formation [45].

Studies aimed at elucidating the spatiotemporal mechanisms of AURKB localization and activation have shown that the binding of INCENP to AURKB partially activates AURKB. INCENP then becomes a substrate of AURKB as AURKB phosphorylates the “IN” box of INCENP. Complete activation of AURKB also requires its own phosphorylation (in trans) in a positive feedback loop. INCENP, Survivin/BIRC5, and Borealin/CDCA8 collaborate to target the CPC to histones in the inner centromere, where the crowding of CPC complexes within coacervates promotes both INCENP phosphorylation and AURKB autophosphorylation (both in trans), and results in the full activation of AURKB [35,46]. The phase separation property of the CPC is also critical for its role in correcting erroneous kinetochore-microtubule connections and in maintaining the SAC [36]. Successful SAC activation and execution of cytokinesis are also dependent on AURKB [37]. If AURKB is mutated or overexpressed, as in TNBC, there is a high risk of improper chromosome segregation [47]. Hyperactivation of AURKB has been shown to result in aneuploidy and increased tumor formation [48]. Overexpression of AURKB in murine mammary epithelial cells drives whole genome duplication and multinucleation arising from cytokinesis failure or mitotic slippage [49,50,51]. Unsurprisingly, AURKB is one of the genes in the CIN70 gene signature; CIN70 genes are genes whose overexpression potently drives chromosomal instability and functional aneuploidy in multiple cancer types [52].

BIRC5/Survivin, a protein that is expressed in G2/M, has long been known to have a pro-survival/anti-apoptotic function in mitosis—hence its name [53]. During mitosis, Survivin localizes to the inner centromere and spindle microtubules, indicating that Survivin may be able to simultaneously regulate apoptosis, chromosome biorientation, and the SAC [54]. Survivin has no known enzymatic activity and is believed to mediate its multifarious roles by associating with diverse partner proteins [55]. Thus, it is unclear exactly how survivin inhibits apoptosis. In cancer cells, survivin is first detected in the G2 phase as part of the CPC in the inner centromere [56]. In mitosis, survivin helps ensure that chromosomes are properly aligned by communicating with SAC tension sensors, and it can also affect mitotic spindle assembly by dampening microtubule dynamics [55,57]. In addition, survivin can direct cytokinesis by mapping the cleavage plane [58]. Previous studies have shown that during mitosis, BIRC5/Survivin exists in a complex with the centrosome clustering protein KIFC1; furthermore, overexpression of KIFC1 led to an increase in the steady-state levels of both AURKB and BIRC5/Survivin [30]. As survivin plays critical roles in decreasing apoptosis and promoting mitotic progression, there are countless routes by which overexpression of survivin can promote tumor cell survival, chemoresistance, and disease progression.

Cell division cycle associated 8 (CDCA8/Borealin/DasraB) was discovered in two independent studies of proteins associated with mitotic chromosomes and was found to also play essential roles in the generation of successful kinetochore-spindle attachments and in maintaining genomic stability as cells traverse mitosis [59,60,61]. CDCA8 is a putative oncogene that is overexpressed in numerous cancer types and is essential for tumor invasion and metastasis [62]; moreover, CDCA8 is widely expressed in embryonic tissues but is absent or expressed at low levels in normal adult cells [63,64,65]. The capacity of CDCA8/Borealin to bind DNA evokes the possibility that CDCA8 mediates the attachment of the CPC complex to the inner centromere [66]. Inactivation of CDCA8 results in polyploidy, stalled mitoses, and problems with normal embryonic tissue development, attesting to CDCA8’s critical roles in cell proliferation [67]. In HeLa cells with depleted amounts of CDCA8, CDCA8 failed to localize to the spindle midzone and delocalized INCENP, AURKB, and BIRC5 [59]. This observation reflects CDCA8’s vital role in ensuring that all CPC components localize correctly and that cells traverse mitosis smoothly. Additionally, CDCA8 phosphorylation is critical for the inner centromere targeting of the CPC in prometaphase, as well as for chromosome biorientation [68].

## 5. Acting in Concert: The Centrosome Clustering Proteins KIFC1, AURKB, BIRC5, and CDCA8 Are Overexpressed in a Variety of Tumor Tissues, Including Breast Cancers

**Figure 1:**
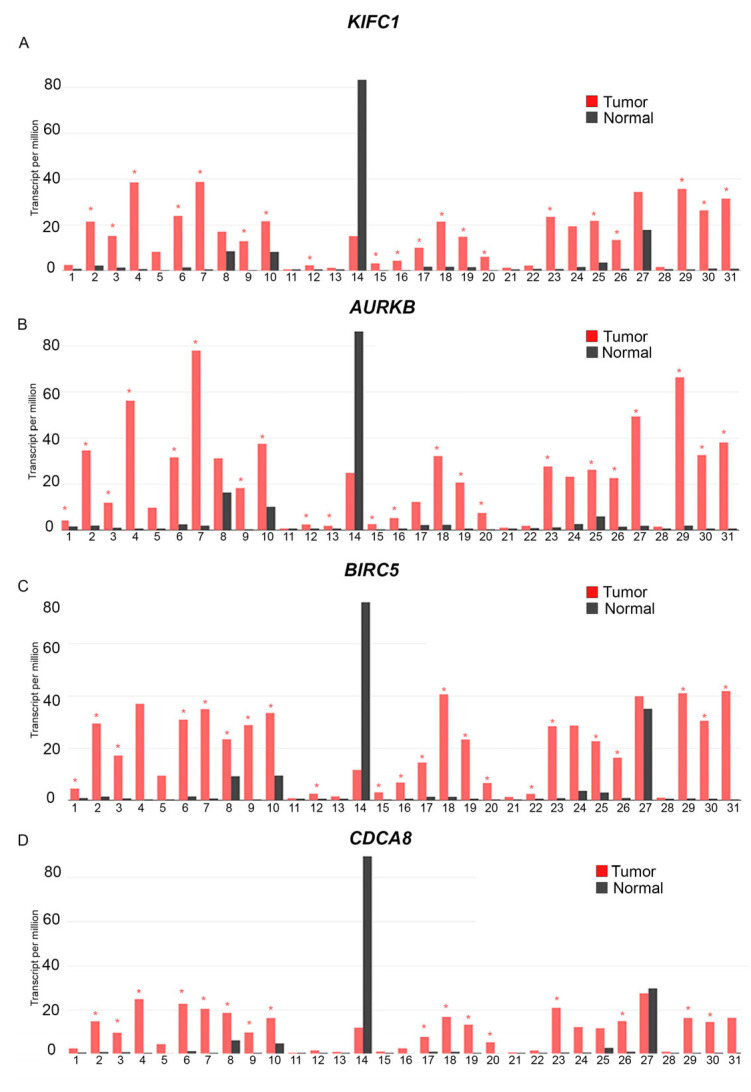
KIFC1, AURKB, BIRC5, and CDCA8 are overexpressed in a variety of cancer types. RNA sequencing data from 9736 tumor and 8587 normal samples were analyzed for the expression profiles of centrosome clustering proteins of interest across diverse cancer types using GEPIA. The red bars represent the expression of (**A**) KIFC1, (**B**) AURKB, (**C**) BIRC5, and (**D**) CDCA8, respectively, in tumor tissues from 31 different cancer types, while the black bars represent the expression of the aforementioned genes in matched normal tissues from the corresponding tumor types. The cancer types wherein the expression of these genes in tumor tissues is significantly higher than their expression in corresponding normal tissues show a red asterisk on the tumor tissue (red) bar (*p* < 0.05; ANOVA test, Ilog2FCI cutoff of 1).

Given that centrosome amplification and clustering are both highly pervasive hallmarks of tumors, we aimed to gain a better understanding of the landscape of centrosome clustering protein expression across diverse cancer types. We analyzed RNA sequencing expression data of 9736 tumors and 8587 normal samples from The Cancer Genome Atlas (TCGA) and the Genotype-Tissue Expression (GTEx) projects for the expression profiles of centrosome clustering proteins KIFC1, AURKB, BIRC5, and CDCA8. Our analyses of TCGA and GTex tumor data and matched normal samples from TCGA and GTex datasets using the Gene Expression Profiling Interactive Analysis (GEPIA) tool [69] showed that KIFC1 (Figure 1A), AURKB (Figure 1B), BIRC5 (Figure 1C), and CDCA8 (Figure 1D) are all significantly overexpressed in many different cancer types.

1: Adenoid Cystic Carcinoma, Tumor (n = 77), Normal (n = 88).
2: Bladder Urothelial Carcinoma, Tumor (n = 404), Normal (n = 28)
3: Breast Invasive Carcinoma, Tumor (n = 1085), Normal (n = 291)
4: Cervical squamous cell carcinoma and endocervical adenocarcinoma, Tumor (n = 306), Normal (n = 13)
5: Cholangiocarcinoma, Tumor (n = 36), Normal (n = 9)
6: Colon adenocarcinoma, Tumor (n = 275), Normal (n = 349)
7: Lymphoid Neoplasm Diffuse Large B-cell Lymphoma, Tumor (n = 47), Normal (n = 337)
8: Esophageal carcinoma) Tumor (n = 182), Normal (n = 286)
9: Glioblastoma Multiforme, Tumor (n = 163), Normal (n = 207)
10: Head and Neck squamous cell carcinoma, Tumor (n = 519), Normal (n = 44)
11: Kidney Chromophobe, Tumor (n = 66), Normal (n =5 3)
12: Kidney renal clear cell carcinoma, Tumor (n = 523), Normal (n = 100)
13: Kidney renal clear cell carcinoma, Tumor (n = 286), Normal (n = 60)
14: Acute Myeloid Leukemia, Tumor (n = 173), Normal (n = 70)
15: Brain Lower Grade Glioma, Tumor (n = 518), Normal (n = 207)
16: Liver hepatocellular carcinoma, Tumor (n = 369), Normal (n = 160)
17: Lung adenocarcinoma, Tumor (n = 485), Normal (n = 347)
18: Lung squamous cell carcinoma, Tumor (n = 486), Normal (n = 338)
19: Ovarian serous cystadenocarcinoma, Tumor (n = 426), Normal (n = 88)
20: Pancreatic adenocarcinoma, Tumor (n = 179), Normal (n = 171)
21: Pheochromocytoma and Paraganglioma, Tumor (n = 182), Normal (n = 3)
22: Prostate adenocarcinoma, Tumor (n =4 92), Normal (n = 152)
23: Rectum adenocarcinoma, Tumor (n = 92), Normal (n = 318)
24: Sarcoma, Tumor (n = 261), Normal (n = 2)
25: Skin Cutaneous Melanoma, Tumor (n = 461), Normal (n = 558)
26: Stomach Adenocarcinoma, Tumor (n = 408), Normal (n = 211)
27: Testicular Germ Cell Tumors, Tumor (n = 137) Normal (n = 165)
28: Thyroid Cutaneous Carcinoma, Tumor (n = 512), Normal (n = 337)
29: Thymoma, Tumor (n = 118), Normal (n = 339)
30: Uterine Corpus Endometrial Carcinoma, Tumor (n = 174), Normal (n = 91)
31: Uterine Carcinosarcoma, Tumor (n = 57), Normal (n = 78)

**Figure 2:**
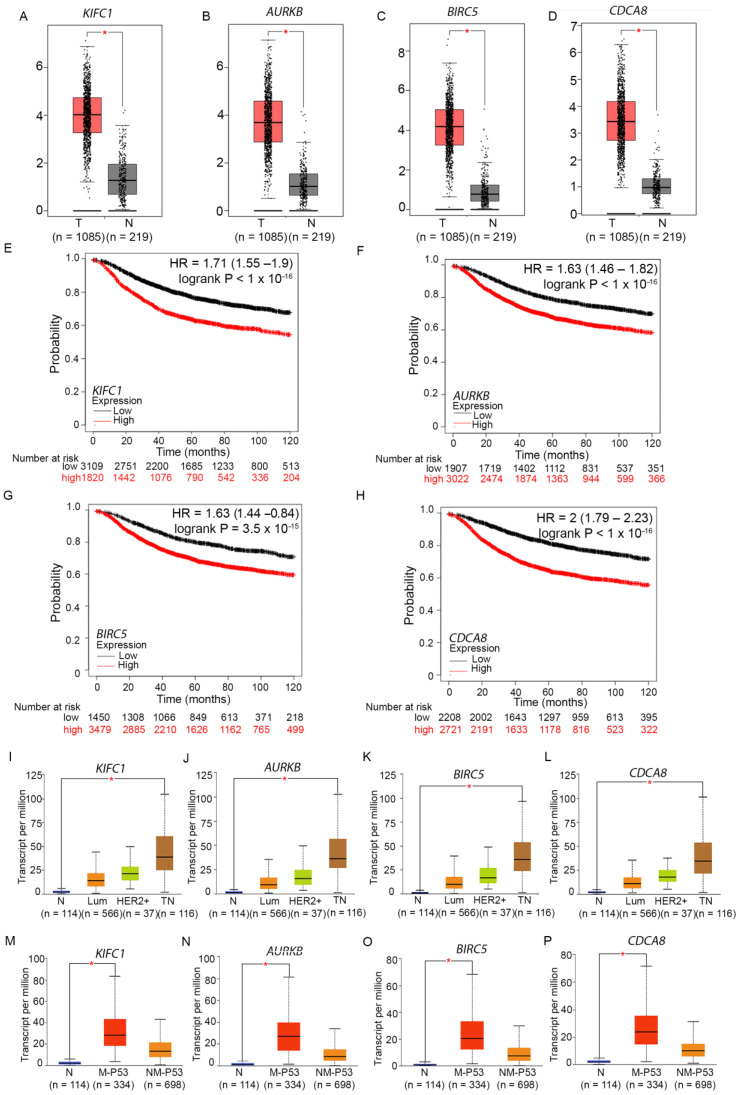
Prognostic significance of centrosome clustering proteins and their association with breast cancer subtypes and TP53 mutation status. (**A**–**D**) Comparison of the expression of centrosome clustering genes in breast tumor tissues versus normal tissues, performed using TCGA RNA sequencing data available on the GEPIA platform. The red box plot represents the expression of (**A**) KIFC1, (**B**) AURKB, (**C**) BIRC5, and (**D**) CDCA8 in breast invasive carcinoma, while the black plot represents the expression in normal breast tissues. Each dot signifies an individual, and their expression falls within a specific category. The red asterisk denotes that the difference between the two samples is statistically significant (*p* < 0.05). (**E**–**H**) Kaplan–Meier Survival analysis to evaluate the prognostic significance of centrosome clustering genes. Microarray data were analyzed using the KM Plotter tool. The red line represents the survival of patients with above-cutoff levels of (**E**) KIFC1, (**F**) AURKB, (**G**) BIRC5, and (**H**) CDCA8 expression in breast tumors, while the black line represents the survival of patients with below-cutoff levels of the same clustering genes (the probe set used for analysis was the Jetset optimal microarray probe set, and the optimal cutoff was used for analysis, with no restriction of subtypes) in relation to recurrence-free survival over the course of 120 months. (**I**–**L**) Breast tumor subtype analysis of the expression of centrosome clustering genes. The expression of (**I**) KIFC1, (**J**)AURKB, (**K**) BIRC5, and (**L**) CDCA8 in breast invasive carcinoma subclasses of the TCGA dataset are shown using box-whisker plots. The red asterisk indicates a statistically significant difference in expression (*p* < 0.05); n = sample size per subtype; N = Normal, Lum = Luminal, HER2+ = Her2-positive tumors, TN = triple-negative. Not shown, but still important, is the significance of overexpression of genes in triple-negative compared to Luminal and HER2+ (KIFC1: Luminal-vs.-TNBC: *p* < 1 × 10^−12^, HER2 Positive-vs.-TNBC: *p* = 3.04 × 10^−8^; AURKB: Luminal-vs.-TNBC: *p* = 1.6 × 10^−12^, HER2 Positive-vs.-TNBC: *p* = 4.9 × 10^−9^; BIRC5: Luminal-vs.-TNBC: *p* = 1.6 × 10^−12^, HER2 Positive-vs.-TNBC: *p* = 2.1 × 10^−5^; CDCA8: Luminal-vs.-TNBC: *p* < 1.0 × 10^−12^, HER2 Positive-vs.-TNBC: *p* = 4.5 × 10^−7^). (**M**–**P**) Analysis of the TCGA dataset (data found on the UALCAN platform) for the expression of centrosome clustering genes based on the TP53 mutation status of breast tumors. Box-whisker plots showing the expression of (**M**) KIFC1, (**N**)AURKB, (**O**) BIRC5, and (**P**) CDCA8 in breast tumors categorized as having mutations in TP53 (M-P53) or having a non-mutant TP53 status (NM-P53). The red asterisk indicates a statistically significant difference in expression (*p* < 0.05); n = sample size per subtype.

We found that all four aforementioned genes were significantly overexpressed in bladder urothelial carcinoma, breast cancer, colon adenocarcinoma, lymphoid neoplasm diffuse large B-cell lymphoma, glioblastoma multiforme, head and neck squamous cell carcinoma, lung squamous cell carcinoma, ovarian serous cystadenocarcinoma, pancreatic adenocarcinoma, rectum adenocarcinoma, stomach adenocarcinoma, thymoma, and uterine corpus endometrial carcinoma tumor tissues compared to matched normal samples. In a previous study, de Almeida et al. [70] quantified CA20 (a gene expression-based score that uses the expression of 20 genes associated with centrosome amplification as a surrogate estimate of the level of centrosome amplification present in various samples) in 9721 tumor and 725 matched normal samples spanning 32 cancer types from TCGA. The authors found that CA20 was higher in tumor versus matched normal samples in 15 different cancer types (False Discovery Rate (FDR) < 0.0001, Wilcoxon rank-sum test), including bladder urothelial carcinoma, breast invasive carcinoma, colon adenocarcinoma, rectal adenocarcinoma, head and neck squamous cell carcinoma, lung adenocarcinoma, lung squamous cell carcinoma, stomach adenocarcinoma, uterine corpus endometrial carcinoma, kidney renal papillary cell carcinoma, prostate adenocarcinoma, thyroid carcinoma, kidney chromophobe, kidney renal clear cell carcinoma, liver hepatocellular carcinoma, and esophageal carcinoma. These findings supported the notion that centrosome amplification is indeed a hallmark of tumors and is widespread. Our data showing a pan-cancer pattern of upregulation of crucial centrosome clustering proteins suggest that there exist potential mechanisms to co-upregulate clustering pathways in conjunction with pathways that result in amplified centrosomes because centrosome amplification can result in the death of cells harboring excess centrosomes if it is not accompanied by a concomitant upregulation of centrosome clustering mechanisms. In breast cancers, we found significant upregulation of KIFC1 (Figure 2A), AURKB (Figure 2B), BIRC5 (Figure 2C), and CDCA8 (Figure 2D) in tumor samples of both the GEPIA (Figure 2A–D) and UALCAN datasets, compared to normal samples.

## 6. Aiding and Abetting: Overexpression of Centrosome Clustering Proteins KIFC1, AURKB, BIRC5, and CDCA8 Is Associated with Poor Prognosis, Triple-Negative Status, and TP53 Mutant Status of Breast Cancers

To examine associations between the expressions of KIFC1, AURKB, BIRC5, and CDCA8 and relapse-free survival, we performed Cox proportional hazards regression analysis for each gene separately, using the Kaplan–Meier Plotter (KM Plotter) online tool [71]. For each gene, each possible cutoff value was examined between the lower and upper quartiles, and the optimal cutoff chosen was the one that yielded the lowest *p*-value for the logrank test. Kaplan–Meier plots were then used to visualize associations between gene expression and survival. Our analyses of publicly available microarray data using the (KM Plotter) tool for breast cancer showed that high levels of expression of KIFC1 (Figure 2E), AURKB (Figure 2F), BIRC5 (Figure 2G), and CDCA8 (Figure 2H) predicted significantly poorer recurrence-free survival of breast cancer patients, suggesting that upregulation of centrosome clustering mechanisms could potentially contribute to disease progression in breast cancer patients.

Interestingly, breast cancer subtype analyses using TCGA RNA sequencing data available on The University of Alabama at Birmingham CANcer data analysis portal (UALCAN) [72,73] platform showed that the expression levels of KIFC1 (Figure 2I), AURKB (Figure 2J), BIRC5 (Figure 2K), and CDCA8 (Figure 2L) were the highest in TNBCs, suggesting their high importance in driving the biology of breast tumors with TN status. Previous experimental work has shown that TNBCs have higher levels of centrosome amplification compared to non-TNBC subtypes and that TNBCs have higher CA20 scores than grade-matched non-TNBCs [32]. These findings suggest that TNBCs, which are well known to exhibit high levels of centrosome amplification, also express centrosome clustering proteins at high levels and that this pattern of co-overexpression of centrosome amplification and clustering genes underlies the higher genomic instability and elevated risk of disease progression observed in TNBCs [30]. This notion was further corroborated by our finding that KIFC1 (Figure 2M), AURKB (Figure 2N), BIRC5 (Figure 2O), and CDCA8 (Figure 2P) are all significantly overexpressed in TP53-mutant breast tumors. TP53 is mutated in 65–80% of TNBCs [74]. Loss or loss-of-function of p53 deregulates the centrosome duplication cycle and precipitates centrosome amplification [75]. TP53 employs both transactivation-dependent and transactivation-independent pathways to control the numerical homeostasis of centrosomes. In its transactivation-dependent control pathway, the CDK inhibitor p21(Waf1/Cip1) acts as a major p53 effector that ensures the tight coordination of centrosome duplication with DNA replication in the S phase. TP53’s direct binding to centrosomes enables TP53’s transactivation-independent suppression of abnormal centrosome amplification [76]. Taken together, our data above suggest that breast tumors, especially triple-negative and TP53-mutant breast tumors, show high levels of co-expression of genes implicated in centrosome amplification as well as genes implicated in centrosome clustering and that the coupling between these two mechanisms could engender poor outcomes in breast cancer patients.

## 7. Building Alliances: Oncogenic Proteins FOXM1, E2F1, and ATAD2 Are Overexpressed in Breast Tumors, Especially Those with Mutant TP53, and Are Associated with a Poor Prognosis

To explore potential molecular mechanisms connecting the upregulation of genes that drive centrosome amplification in breast tumors to the upregulation of genes that mediate centrosome clustering, we performed a detailed study of the relevant literature. We found several studies that indicated that KIFC1, AURKB, BIRC5, and CDCA8 were all direct transcriptional targets of FOXM1, an oncogenic Forkhead transcription factor referred to as a master regulator of a network of genes essential for mitotic progression, DNA repair, chromatin assembly, and protein degradation [77]. FOXM1 is a potent driver of tumor metastasis in multiple cancer types [78,79]. Among the transcriptional targets of FOXM1, we found several genes involved in centrosome amplification (AURKA, CCNA2, CDK1, CEP152, PLK1, PLK4, SASS6, and STIL), which suggested that FOXM1 might facilitate the co-expression of centrosome amplification as well as centrosome clustering transcriptional networks—a postulate we explored in greater depth in the analyses described below. Our literature study also revealed that E2F1 is a critical upstream regulator of FOXM1 expression [80]. This finding piqued our interest because E2F1 is a known driver of breast cancer metastasis and angiogenesis and a promoter of disease progression in several cancers [81]. E2F1 is, additionally, a well-established driver of centrosome amplification and is one of the 20 genes whose expression is computed to yield the CA20 score as a surrogate measure of the level of centrosome amplification found in a tumor [32].

We also came across studies that implicated the ATPase family AAA-domain containing protein ATAD2/ANCCA as an emerging oncogene and E2F1 coactivator that functions at all transcriptionally active regions of the genome starting in the G1/S phase until the onset of the M phase [82]. ATAD2 transcription is initially driven by E2F1; subsequently, ATAD2 participates in epigenetic decoding, transcriptional activation of E2F-target genes, and oncogenic signaling via c-myc [83]. ATAD2 levels are low or absent in healthy somatic cells, but ATAD2 is upregulated in diverse cancer types, wherein high ATAD2 expression is associated with a high histological grade, high rates of metastasis and recurrence, and poor overall survival [84,85].

**Figure 3:**
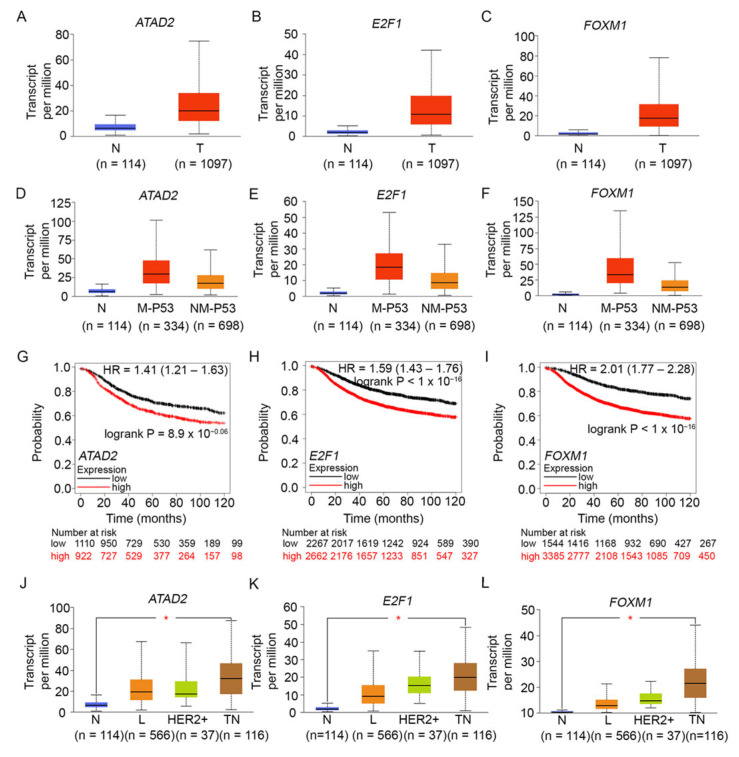
Analysis of the expression levels and prognostic significance of ATAD2, E2F1, and FOXM1 oncogenes in breast tumors. (**A**–**C**) Comparison of expression of the oncogenes (**A**) ATAD2, (**B**) E2F1, and (**C**) FOXM1 in breast tumor tissues versus normal tissues, performed using TCGA RNA sequencing data available on the UALCAN platform. The red box plot represents gene expression level in breast invasive carcinoma, while the blue plot represents gene expression in matched normal breast tissues. The red asterisk indicates a statistically significant difference in expression (*p* < 0.05). (**D**–**F**) Analysis of the TCGA dataset (data found on the UALCAN platform) for the expression of specific oncogenes of interest based on the TP53 mutation status of breast tumors. Box-whisker plots showing the expression of (**D**) ATAD2, (**E**) E2F1, and (**F**) FOXM1 in breast tumors categorized as having mutations in TP53 (M-P53) or having a non-mutant TP53 status (NM-P53). The red asterisk indicates a statistically significant difference in expression (*p* < 0.05); n = sample size per subtype. (**G**–**I**) Kaplan–Meier Survival analysis to evaluate the prognostic significance of our oncogenes of interest in breast cancer. Microarray data were analyzed via the KM Plotter tool. The red line represents the survival of patients with above-cutoff levels of (**G**) ATAD2, (**H**) E2F1, and (**I**) FOXM1 expression in breast tumors, while the black line represents the survival of patients with below-cutoff levels of the same oncogenes (the probe set used for analysis was the Jetset optimal microarray probe set, the optimal cutoff was used for analysis, and no restriction of subtypes) in relation to recurrence-free survival over the course of 120 months. (**J**–**L**) Breast tumor subtype analysis of the expression of specific oncogenes of interest. The expression of (**J**) ATAD2, (**K**) E2F1, and (**L**) FOXM1 in breast carcinoma subclasses of the TCGA dataset are shown using box-whisker plots. The red asterisk indicates a statistically significant difference in expression (*p* < 0.05); n = sample size per subtype; N = Normal, Lum = Luminal, HER2+ = Her2-positive tumors, TN = triple-negative. *p*-values for other statistically significant differences were: ATAD2: Normal-vs.-TNBC: 1.62 × 10^−12^, Luminal-vs.-TNBC: 6.06 × 10^−5^, HER2 Positive-vs.-TNBC: *p* = 7.5 × 10^−3^; E2F1: Normal-vs.-TNBC: <1 × 10^−12^, Luminal-vs.-TNBC: 1.35 × 10^−11^; FOXM1: Normal-vs.-TNBC: 1.62 × 10^−12^, Luminal-vs.-TNBC: 1.62 × 10^−12^, HER2 Positive-vs.-TNBC: 4.05 × 10^−05^. Other values that were statistically relevant are: ATAD2: Normal-vs.-Luminal: <1 × 10^−12^, Normal-vs.-HER2 Positive: 1.35 × 10^−06^; E2F1: Normal-vs.-Luminal: <1 × 10^−12^, Normal-vs.-HER2 Positive: 1.69 × 10^−10^, Luminal-vs.-HER2 Positive: 9.32 × 10^−03^;FOXM1: Normal-vs.-Luminal: 1.62 × 10^−12^, Normal-vs.-HER2 Positive: 2.54 × 10^−07^, Luminal-vs.-HER2 Positive: 1.29 × 10^−02^.

To better understand the connections between ATAD2, E2F1, and FOXM1 and the expression of our centrosome clustering genes of interest, we performed additional in silico analyses of publicly available gene expression datasets. Analysis of TCGA RNA sequencing data through the UALCAN platform revealed that the expression of ATAD2 (Figure 3A), E2F1 (Figure 3B), and FOXM1 (Figure 3C) were all significantly higher in primary breast tumor samples when compared to normal samples. Furthermore, ATAD2 (Figure 3D), E2F1 (Figure 3E), and FOXM1 (Figure 3F) were all significantly overexpressed in breast tumors harboring mutant TP53 compared to breast tumors harboring non-mutant TP53. This finding was similar to our earlier data showing that the centrosome clustering genes KIFC1, AURKB, BIRC5, and CDCA8 were all upregulated in TP53-mutant breast tumors. We then confirmed our findings using the muTarget tool [86] to investigate the effect of mutations in the TP53 coding region (i.e., our input genotype) on downstream gene expression in a sample set comprising 305 TP53-mutant and 674 TP53-wild-type breast cancers. In this dataset, KIFC1 showed a 1.95-fold upregulation (*p* = 5.36 × 10^−41^), AURKB showed a 2.35-fold upregulation (*p* = 1.36 × 10^−48^), BIRC5 showed a 1.97-fold upregulation (*p* = 1.41 × 10^−42^), CDCA8 showed a 2.23-fold upregulation (*p* = 5.11× 10^−55^), ATAD2 showed a 1.63-fold upregulation (*p* = 6.23 × 10^−23^), E2F1 showed a 1.77-fold upregulation (*p* = 2.55 × 10^−32^), and FOXM1 showed a 2.37-fold upregulation (*p* = 1.95 × 10^−45^), in TP53-mutant versus TP53-wild-type breast cancers. These data compellingly indicate that TP53-mutant breast tumors show a significant upregulation of KIFC1 and centrosome clustering CPC genes, as well as a concomitant overexpression of ATAD2, E2F1, and FOXM1. To examine associations between the expressions of FOXM1, ATAD2, and E2F1 and relapse-free survival, we performed Cox proportional hazards regression analysis for each gene separately, using the KM Plotter online tool. For each gene, each possible cutoff value was examined between the lower and upper quartiles, and the optimal cutoff chosen was the one that yielded the lowest *p*-value for the logrank test. Kaplan–Meier plots were used to visualize the associations between gene expression and survival. Analysis of microarray data using the KM Plotter tool showed that overexpression of ATAD2 (Figure 3G), E2F1 (Figure 3H), and FOXM1 (Figure 3I), each conferred significantly poorer recurrence-free survival in breast cancer patients. We then used the KM plotter tool to design a weighted gene signature that uses the mean expression of FOXM1, ATAD2, and E2F1 (weighted as 1.0, 0.8, and 2.3, respectively) and utilizes the optimal cut-point to stratify TNBC patients in the dataset (n = 220 samples); we found that above-cutoff expression of this oncogene triad signature predicted poorer recurrence-free survival in TNBC patients (HR = 1.68; 95% CI: 1.00–2.82; *p*-value for the logrank test = 0.047). We also found that AURKB, BIRC5, CDCA8, and FOXM1 are all part of the meta-PCNA proliferation gene signature, and high levels of expression of genes in this signature are associated with poor prognosis in breast cancer [87]. Collectively, our data from the analyses described above suggested that overexpression of this trio of oncogenic proteins showed a pattern very similar to the overexpression of centrosome clustering genes KIFC1, AURKB, BIRC5, and CDCA8 and similarly conferred poorer prognosis in patients with breast cancer, especially TP53-mutant breast cancers.

Analysis of RNA sequencing data in the UALCAN portal also showed that the expression levels of ATAD2 (Figure 3J), E2F1 (Figure 3K), and FOXM1 (Figure 3L) oncogenes were the highest in TNBCs among breast cancer subtypes, suggesting that the simultaneous overexpression of (i) ATAD2, E2F1, and FOXM1; (ii) centrosome amplification genes; and (iii) the centrosome clustering genes KIFC1, AURKB, BIRC5, and CDCA8 could play a key role in the tumor biology of TNBC. Our analysis of TNBC gene expression data from TCGA (performed on the UALCAN platform) yielded more data that lent credence to this idea. We found that (a) FOXM1 and AURKB were among the top 25 genes overexpressed among TNBCs, (b) BIRC5 was among the top 26–50 genes overexpressed among TNBCs, (c) KIFC1 and CDCA8 were among the top 51–75 genes overexpressed among TNBCs, (d) E2F1 was among the top 101–125 genes overexpressed among TNBCs, and (e) ATAD2 was among the top 151–175 genes overexpressed in TNBCs.

## 8. A Team Effort: CPC Genes Interact with p53 in Distinct Ways

As the “guardian of our genome,” p53 is indeed a tour de force that galvanizes the expression of target genes to induce cell cycle arrest, senescence, repair of damaged DNA, stress response, or even cell death, as appropriate, and all of these pathways contribute to p53’s renowned tumor suppressor function. TP53 is the most frequently mutated gene in cancer, and more than 45,000 somatic and germline TP53 mutations have been collated (http://p53.fr). Given that overexpression of KIFC1, as well as the CPC proteins involved in centrosome clustering, was associated with a p53 mutant status of breast tumors, we delved deeper into the literature to uncover known connections between these clustering proteins and p53 function. Our study of the literature revealed that AURKB has also been demonstrated to phosphorylate the tumor suppressor p53 at S183, T211, and S215 during interphase, and to enhance the ubiquitin-dependent degradation of p53, thus suppressing the expression of p53 target genes such as the CDK inhibitor, p21 [88]. It has also been reported that AURKB directly interacts with p53 at the CPC during mitosis and that the two proteins colocalize throughout various phases of mitosis (prometaphase, metaphase, anaphase, and telophase). Marxer et al., 2014 also found that cancer cells lacking p53 expression became sensitized to AURKB inhibitors [89]. Given the role of AURKB in regulating steady-state levels of p53, it is plausible that oncogenic overexpression of AURKB may severely compromise the stability and tumor suppressor function of p53 [88].

While BIRC5/survivin has been known to regulate chromosome segregation independently of p53, most of the previous literature seems to agree that survivin also often participates in a p53-dependent apoptotic pathway and is negatively regulated by wild-type p53 in some manner [90]. Researchers have largely pinpointed two main mechanisms by which BIRC5 expression is regulated by p53. First, Mirza et al., 2002, after investigating an ovarian carcinoma cell line, suggested that instead of directly binding to the BIRC5 promoter, p53 inhibits an acetylase enzyme, thus preventing the acetylation of the BIRC5 promoter [91]. If the chromatin is not acetylated, BIRC5 will not be transcribed. If p53 is mutated, then the BIRC5 promoter can be acetylated, allowing the required transcription factors to bind and promote BIRC5 expression. On the other hand, Hoffman et al., 2002 suggest that p53 binds directly with the BIRC5 promoter to repress BIRC5 expression in a lung adenocarcinoma cell line [92]. They came to this conclusion after finding that, with functional p53, the survivin promoter was sufficient to repress survivin transcription in transfected cells. In another study using human melanocytes, Raj et al., 2007 found that direct binding of p53 to the BIRC5 promoter occurs in normal cells and that there is increased promoter activity when the p53-binding site is mutated, thus supporting Hoffman et. al.’s findings [93].

Reports in the previous literature suggest that CDCA8 regulates cell cycle progression in conjunction with p53, although further studies are required to shed light on the mechanistic basis of this regulation. In endometrial cancer, the overexpression of CDCA8 resulted in decreased levels of p53 and Rb; in contrast, CDCA8 knockdown resulted in an increase in p53 and Rb levels, implying that CDCA8 plays a role in regulating the steady-state levels of these proteins in endometrial cancer and likely regulates the activation of pathways downstream of p53/Rb [94]. High levels of CDCA8 also stimulate breast cancer progression and fuel a notable downregulation of the p53 target p21 in breast cancer [66]. Date et al., 2007 found that CDCA8 is overexpressed in many brain, colon, and lymphoma cancers (45% of brain cancers, 34% of lymphomas, and 61% of colon cancers had at least 50% higher levels of CDCA8/Borealin compared to healthy tissue) [95]. They found that CDCA8/Borealin expression was repressed by high levels of p53 in human colorectal carcinoma cell lines, and Rb family proteins are involved in the downregulation of CDCA8 in response to high p53 signaling [89]; however, the authors noted that p53 was not the sole factor regulating CDCA8 expression and that other unknown transcription factors were likely also involved [95].

To explore the idea that p53 may modulate the transcription of our target centrosome clustering genes, we ran an analysis of their promoter regions and identified p53 binding sequences. To do this, we downloaded the genomic sequence 1000 base pairs upstream of the transcription start site of AURKB, BIRC5/survivin, and CDCA8/borealin. We then input these sequences into PROMO TF Bind—a tool that utilizes transcription factor binding sites in the TRANSFAC database [96] to construct specific binding site weight matrices for transcription factor binding site prediction [97,98]. This online tool generated a list of transcription factor binding sites found in the promoters of the centrosome clustering genes of interest, with less than 15% dissimilarity from each respective consensus sequence and a random expectation value (a measure of the reliability of the hit) of less than 1.5. We found 18 potential p53 binding half-sites in BIRC5/survivn’s promoter region, 24 in that of CDCA8/borealin, and 15 in that of AURKB. The tumor suppressor p53 is known to bind to a nucleosomal target sequence, which means that the presence of p53 binding sites does not automatically imply that p53 binds to those sites. We are also cognizant of the fact that p53’s ability to bind promoter sequences to regulate transcription is impacted by its mutational status and load [99], its oligomerization state [100], the posttranslational modifications it bears [101], and other proteins it interacts with [102,103]. While it additionally remains to be experimentally determined whether the p53 binding sites we found to have the requisite genomic context, nucleosomal architecture, or a chromatin state favorable for actually binding p53, our data serve as a starting point for investigating if p53 potentially regulates transcription of centrosome clustering genes through direct promoter binding.

## 9. Peas in a Pod: FOXM1, ATAD2, E2F1, and Centrosome Clustering Genes Are Co-Upregulated among AR-Low TNBCs

**Figure 4:**
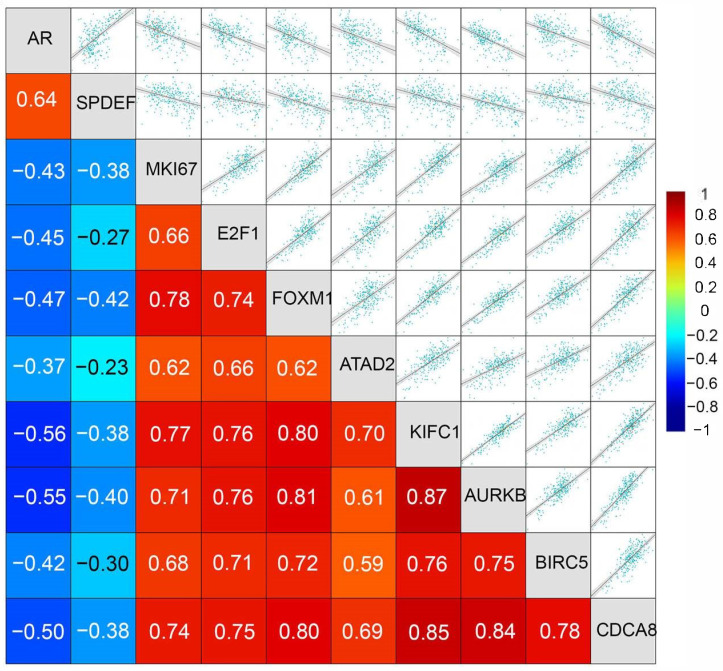
Analysis of correlations between expression of key centrosome clustering genes and expression of their potential upstream regulators and downstream targets in TNBC. bc-GenExMiner’s “targeted” gene correlation analysis of 10 genes (all RNA sequencing data, TNBC status determined by immunohistochemistry). Scatter plots depict Pearson’s pairwise correlations, and the numbers inside the squares indicate the strength of the observed Pearson’s pairwise correlations. Total n = 4421 for each pairwise comparison. Strong negative correlations are depicted in blue, and strong positive correlations are depicted in warm colors. *p*-values for all pairwise correlations were statistically significant (*p* < 0.0001).

Given that TNBCs exhibit rampant centrosome amplification and, therefore, have an elevated need for robust expression of centrosome clustering proteins, we decided to focus our investigation on factors crucial for co-upregulating the expression of centrosome amplification and clustering mechanisms among TNBCs. Using the “Targeted Correlation Analysis” tool of bc-Genexminer [104], we observed strong positive correlations between the expressions of ATAD2, E2F1, FOXM1, KIFC1, AURKB, BIRC5, and CDCA8 among TNBCs (Figure 4), showing that common mechanisms could potentially underlie their coordinated expression.

**Figure 5:**
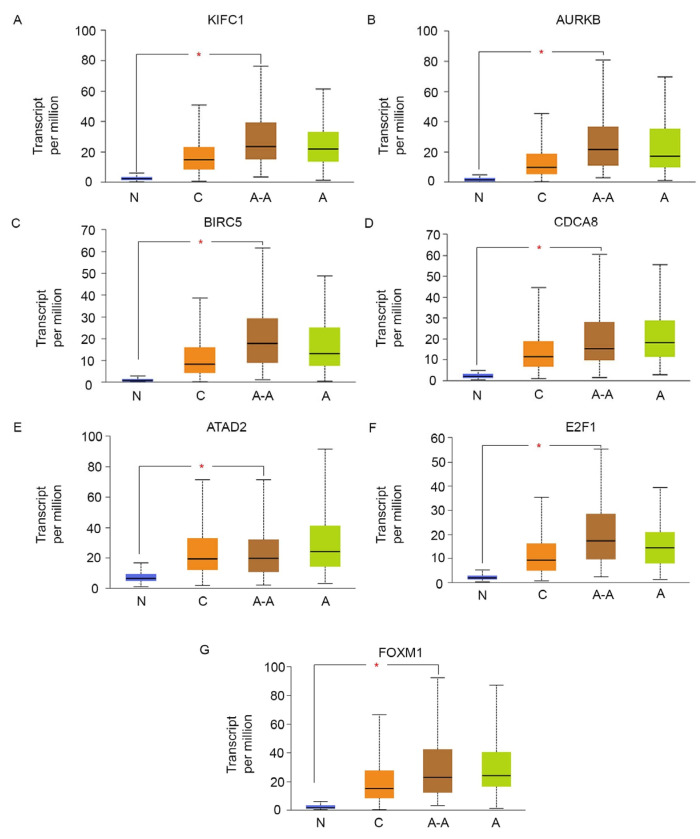
Analysis of the expression levels of ATAD2, E2F1, and FOXM1 oncogenes in breast tumors of patients of different races. (**A**–**G**) Box-whisker plots showing the expression levels of (**A**) KIFC1, (**B**) AURKB, (**C**) BIRC5, (**D**) CDCA8, (**E**) ATAD2, (**F**) E2F1, and (**G**) FOXM1 in breast tumors from patients of different races (self-identified). “N” represents normal breast tissues with a sample size, n = 114 for all, “C” represents Caucasians with a sample size, n = 748, “A-A” represents African Americans with a sample size, n = 179, and “A” represents Asians with a sample size, n = 61. Analysis of TCGA RNA sequencing data was performed using the UALCAN platform. The red asterisk indicates a statistically significant difference in expression levels between the indicated groups (*p* < 0.05). Listed below are the *p*-values proving statistical significance for the data that compares expression with African-American subjects, (KIFC1 race: Normal-vs.-African-American: <1 × 10^−12^, Caucasian-vs.-African-American: 4.84 × 10^−08^; AURKB race: Normal-vs.-African-American: 1.62 × 10^−12^, Caucasian-vs.-African-American: 2.61 × 10^−11^, African-American-vs.-Asian: 2.27 × 10^−02^; BIRC5 race: Normal-vs.-African-American: <1 × 10^−12^, Caucasian-vs.-African-American: 7.72 × 10^−09^; CDCA8: Normal-vs.-African-American: <1 × 10^−12^, Caucasian-vs.-African-American: 1.89 × 10^−03^; ATAD2 race: Normal-vs.-African-American: <1 × 10^−12^, African-American-vs.-Asian: 2.16 × 10^−02^; E2F1 race: Normal-vs.-African-American: <1 × 10^−12^, Caucasian-vs.-African-American: 5.8 × 10^−11^; FOXM1 race: Normal-vs.-African-American: 1.62 × 10^−12^, Caucasian-vs.-African-American: 1.09 × 10^−02^).

The expression of the aforementioned genes was also strongly and positively correlated with the expression of MKI67 (Figure 4), a well-established marker of proliferation in tumors, suggesting that overexpression of this group of oncogenes and centrosome clustering gene, is accompanied by an increase in cell proliferation in TNBC. Interestingly, we found that among TNBCs, the expression of ATAD2, E2F1, FOXM1, and the centrosome clustering genes showed a highly significant negative correlation with the expression of AR as well as a downstream target gene of AR, named SPDEF (Figure 4). This finding suggests that AR-low TNBCs, in particular, show a high expression of ATAD2, E2F1, FOXM1, MKI67, and the centrosome clustering machinery. Since AR-negative TNBC is more commonly diagnosed among African-American women and is believed to underlie the stark racial disparity in breast cancer outcomes in the US [105], we examined the race-wise expression of the centrosome clustering genes KIFC1 (Figure 5A), AURKB (Figure 5B), BIRC5 (Figure 5C), and CDCA8 (Figure 5D), as well as the race-wise expression profiles of ATAD2 (Figure 5E), E2F1 (Figure 5F), and FOXM1 (Figure 5G). We found that almost all of the aforementioned genes showed a significantly higher expression level among African-American breast cancer patients compared to Caucasian/White breast cancer patients. Taken together, our analyses suggest that among AR-low TNBCs, key centrosome clustering genes are co-upregulated alongside centrosome amplification drivers and an oncogenic network that includes ATAD2, E2F1, and FOXM1.

Having compiled all this information, we revisit our central question: what mechanisms co-upregulate the centrosome amplification genes with the centrosome clustering genes, specifically the CPC genes? Here, we identify several mechanisms that couple the expression of these two gene sets and could potentially explain their synchronous overexpression in TNBCs lacking functional p53 and/or AR expression.

## 10. Putting the Puzzle Pieces Together: A Core Transcriptional Network Regulates the Expression of Centrosome Clustering Genes

### 10.1. FOXM1: The Heart and Hub of the Transcriptional Network Controlling G2/M Genes

Forkhead Box M1 (FOXM1) is an oncogenic transcription factor that is upregulated in many cancer types, including breast cancer, where it promotes tumorigenesis and disease progression. FOXM1 is expressed only in proliferating normal and tumor cells [106,107]. The overexpression of FOXM1 in many human cancers is associated with advanced tumor stage, high proliferation rate, aneuploidy, and poor prognosis, as FOXM1 has been experimentally shown to regulate apoptosis, drug resistance, DNA damage repair, stem cell renewal, angiogenesis, metastasis, and mitotic spindle maintenance [108,109,110,111]. As the CPC performs similar roles in the cell cycle, researchers have investigated whether CPC genes are downstream targets of FOXM1. Using quantitative chromatin immunoprecipitation (ChIP) and expression assays, Wang et al., 2005 showed that FoxM1 is essential for the transcription of the mitotic regulatory genes AURKB, BIRC5/survivin, and CDCA8/Borealin [76]. In G2/M, FOXM1 binds to promoters and transactivates AURKB, CDCA8, and BIRC5/survivin, thereby promoting the proliferation of TNBC cells [112,113]. FOXM1 mRNA and protein levels have been found to be significantly elevated in tumors with FOXM1 amplification, p53 inactivation, and Rb-E2F deregulation [114].

### 10.2. DREAM and RB; Engines Controlling Timely Expression of G1/S and G2/M Genes

The dimerization partner, RB-like, E2F, and multi-vulval class B (DREAM) complex, and the retinoblastoma (RB) families of proteins are active throughout many stages of the cell cycle and are primarily responsible for regulating cell cycle-dependent gene expression [115]. At G0, the cell’s resting phase, the p53-p21 pathway is active [116]. Through this pathway, p53 induces the expression of the CDK inhibitor p21 [116]. Owing to the absence of CDK activity, Rb-related proteins p130 and p107 are hypophosphorylated and are able to recruit other members of the DREAM complex, leading to the repression of G2/M genes. Rb itself remains hypophosphorylated and tightly bound to activator E2Fs, resulting in the repression of G1/S genes [117]. In this manner, DREAM and RB coordinately halt entry into the G1 phase of the cell cycle. In the event that p53 has a loss-of-function mutation or is absent, the cell would exit the G0 arrest prematurely.

Cell cycle-regulated genes are broadly categorized into two groups: G1/S regulator genes and G2/M regulator genes. Each of these gene sets is characterized by their distinct promoter DNA motifs and distinct roles. The expression of G1/S genes typically precedes DNA synthesis, and these genes contain an E2F binding motif in their promoters. As such, G1/S genes are regulated primarily by the RB-E2F complex. ATAD2, an E2F coactivator and epigenetic decoder, is also recruited to the G1/S gene promoters, where ATAD2 remodels chromatin architecture to favor the assembly of transcriptionally active complexes and the production of histone modifications that stimulate the transcription of G1/S genes. Conversely, G2/M regulator genes function in mitosis, spindle assembly, chromosome segregation, and cytokinesis (the CPC genes discussed above typically belong to this category). These genes contain a cell cycle gene homology region (CHR) in their promoters and are primarily regulated by DREAM.

While both DREAM and RB operate in G1/S, entry into the cell cycle is primarily regulated by RB at this early stage. In G0/quiescence, RB represses target genes by binding to and inhibiting activator E2F genes (E2F1, E2F2, and E2F3a), which are bound to the E2F promoter regions of target genes. In early G1, Cyclin D mono-phosphorylates RB. However, this hypophosphorylated RB remains bound to the activator E2F complex. In the late G1 to early S phase, Cyclin E and CDK2 fully hyper-phosphorylate RB, essentially inactivating the protein, allowing for the peak expression of G1/S genes [118].

**Figure 6:**
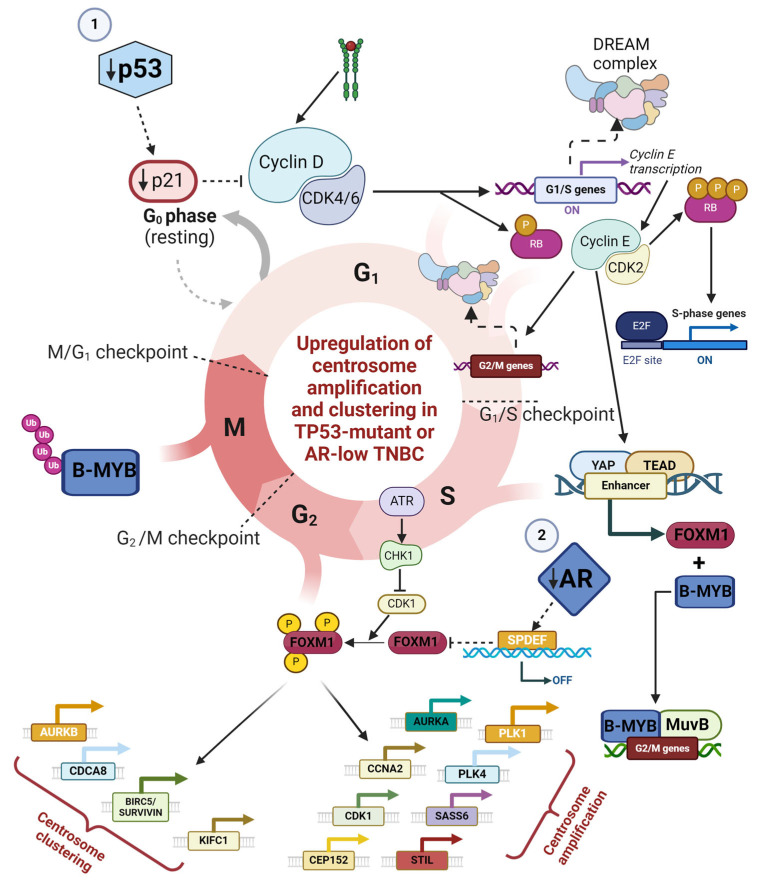
Model depicting how the loss of TP53 function and/or AR expression in TNBC dysregulates a core transcriptional network surrounding the oncogene FOXM1. While both DREAM and RB operate in G1/S, entry into the cell cycle is primarily regulated by RB at this early stage because RB binds and inhibits the activator E2Fs that drive G1/S gene expression. Normally, p53 helps maintain G0 arrest by inducing the expression of CDK inhibitor p21 and maintaining low CDK activity, due to which Rb-related proteins p130 and p107 are hypophosphorylated and are able to recruit other members of the DREAM complex to repress G2/M genes. In the absence of p53 and p21 functions, as depicted in (1), mitogen stimulation increases Cyclin D-CDK4/6 activity that results in the mono-phosphorylation of RB and low-level disassembly of repressive DREAM complexes from the promoters of G1/S genes. An increase in transcription of the G1/S gene Cyclin E then ensues, causing a rise in Cyclin E-CDK2 activity and the onset of centrosome amplification in the cell. Cyclin E-CDK2 then hyperphosphorylates and inactivates RB completely, culminating in peak G1/S gene expression. Loss-of-function of p53 leads to overexpression of oncogenes E2F1 and ATAD2 and could lead to abnormally high Cyclin E-CDK2 activity that causes premature disassembly of DREAM from promoters of G2/M genes (that are extremely sensitive to DREAM perturbation). High Cyclin E-CDK2 activity stimulates YAP/TEAD-mediated transcription of B-MYB and FOXM1, and ATAD2 overexpression also contributes to the build-up of B-MYB. B-MYB combines with MuvB to form the MMB complex at the promoters of G2/M genes, and long-range interactions between MMB at promoter sites and YAP/TEAD at enhancer sites lead to an excessive accumulation of FOXM1 at the promoters of G2/M genes at the S/G2 transition. The phosphorylation and activation of FOXM1 at these promoter sites is restrained throughout the S phase due to ATR-CHK1 inhibition of CDK1. This inhibition is lifted at the end of the S phase, allowing CDK1 to phosphorylate FOXM1. PLK1 also phosphorylates FOXM1, leading to the latter’s full activation at the G2/M boundary. As cells enter the M phase with an excessive amount of FOXM1, B-MYB is degraded and FOXM1 target genes are overexpressed, resulting in the overexpression of several drivers of centrosome amplification (i.e., AURKA, CCNA2, CDK1, CEP152, PLK1, PLK4, SASS6, STIL) and clustering (i.e., including KIFC1, AURKB, BIRC5/Survivin, CDCA8), proliferation, chemoresistance, and apoptosis resistance. In TNBCs, AR normally induces the expression of SPDEF, which constrains FOXM1 levels by disrupting a positive feedback loop that amplifies FOXM1 expression. This constraint is normally reinforced by the p53-p21-DREAM pathway. However, when AR is underexpressed (e.g., in AR-low TNBCs) or not expressed, as depicted in (2), FOXM1 expression is prematurely activated and strongly dysregulated. The nexus of dysregulation surrounding FOXM1 thus drives an aggressive clinical course and a poor prognosis in TP53-mutant and AR-low TNBC.

In quiescent cells, transcription factors B-MYB and FOXM1 undergo cell cycle-regulated proteolysis, and their renewed transcription is repressed by the DREAM complex [119,120]. In G1/S, the DREAM complex is phosphorylated by Cyclin E/CDK2 and dissociates from the promoters of G2/M genes [121]. The dissociation of the DREAM complex is concomitant with the transcription of B-MYB and the formation of MMB (composed of MuvB and B-MYB) at the promoters of G2/M genes. Interestingly, ATAD2 drives B-MYB expression in TNBC cells [122]. The Yes-associated protein 1 (YAP), another transcription cofactor, also promotes the renewed transcription of B-MYB and FOXM1 in G1 and S phases [123,124,125] while simultaneously promoting the assembly of MMB at G2/M CHR sites [126]. Throughout the S phase and DNA replication, G2/M gene expression is repressed by the ATR-CHK1 pathway [124]. Essentially, ATR and CHK1 inhibit the kinase activity of CDK1, allowing the complex of MMB and the accumulated FOXM1 to remain intact [127]. In the G2/M phase, the activation of CDK1 and the consequent lifting of ATR-CHK1 repression allows FOXM1 phosphorylation by CDK1. In turn, this activates the MMB: FOXM1 complex and is soon followed by ubiquitination-dependent degradation of B-MYB. Acetylation of FOXM1 then stabilizes it [128]. Thus, phosphorylated and acetylated FOXM1 and MuvB collaborate to drive strong G2/M gene expression. FOXM1 protein is also known to bind to its own promoter and drive its own expression through positive feedback [129]. Both B-MYB and FOXM1 undergo ubiquitination-dependent proteolysis in a cell cycle-regulated manner: B-MYB is degraded during G2/M (Figure 6), but FOXM1 degradation occurs during exit from mitosis and is Anaphase Promoting Complex (APC)/cyclosome-dependent.

### 10.3. MuvB-FOXM1: Coupling the Expression of Centrosome Amplification Genes and Centrosome Clustering Genes

Our analyses corroborate the idea that perturbations in the regulation of G2/M genes by DREAM lead to a marked shift away from quiescence toward a proliferative cell state characterized by the overexpression of MuvB-FOXM1-driven downstream genes; these downstream genes include both those that stimulate centrosome amplification as well as those that facilitate clustering. Previous bioinformatics analyses of genome-wide DREAM complex-binding data, p53-dependent mRNA expression data, and genome-wide identification of conserved CHR promoter sequences led to the identification of 210 target genes regulated by MuvB-FOXM1 [115]. Among these 210 target genes are (a) several centrosome clustering genes—KIFC1, AURKB, BIRC5, CDCA8, INCENP, CDCA5, CENPA, CKAP5/ch-TOG, MAD2L1, SGOL1, NDC80, and SPC25; (b) the oncogenes FOXM1, B-MYB, and ATAD2; (c) the CA20 centrosome amplification-driving genes AURKA, CCNA2, CDK1, CEP152, PLK1, PLK4, SASS6, and STIL; (d) genes that comprise the proliferation-related 21-gene Oncotype Dx signature that have the ability to predict risk of 10-year distant recurrence in patients with ER+ and axillary lymph node-negative breast cancer—MKI67, AURKA, BIRC5, CCNB1, B-MYB, and (d) MKI67, a marker of proliferation. MuvB-FOXM1 thus couples the expression of several centrosome amplification and centrosome clustering genes and drives their co-upregulation as part of a highly proliferative cell state. The expression of these genes promotes persistent CIN, generation of intratumoral heterogeneity, chemoresistance, and poor outcomes. Several of the MuvB-FOXM1 target genes are also part of the CIN25 and CIN70 gene signatures, whose high expression levels typify aneuploid tumors. Jiao et al., 2015 showed that CDCA8 was frequently overexpressed in breast cancer, and increased expression of CDCA8 was positively associated with FOXM1 expression, triple-negative phenotype, and shorter overall survival. Moreover, the authors found that a combination of CDCA8 and FOXM1 expression showed a higher hazard ratio than the individual markers [130].

### 10.4. The TP53-p21-DREAM Pathway Reins in MuvB-FOXM1

The gene encoding the CDK inhibitor p21 was the first p53 target gene to be identified [131], and p21 inhibits the activity of CDK2 and CDK1 [115]. When CDK activity is inhibited, Rb-related proteins p130 and p107 are hypophosphorylated. Together, these proteins recruit the other members of the DREAM complex, which in turn represses the expression of G2/M genes. P21 is also required for the binding of the transcriptional repressor DREAM complex to the promoters of G2/M genes upon p53 activation [115]. A study by Pfister et al., 2018 that aimed to identify drivers of aneuploidy in breast tumors found that (i) p53 was mutated in most aneuploid tumors, (ii) p53 mutations co-associate with overexpression of several mitotic transcription factors, and that (iii) in these tumors, and across all breast cancer subtypes, the oncogenes E2F1, B-MYB, and FOXM1 drive the overexpression of a network of mitotic proteins that ultimately diminishes the fidelity of chromosome segregation [132]. Importantly, it can be concluded that the p53 mutations per se are unlikely to cause aneuploidy; instead, a “two-hit model” was proposed for generating aneuploidy wherein they ascribe a causative role for the overexpression of FOXM1, B-MYB, and E2F1 in lessening the fidelity of chromosome segregation and producing lagging chromosomes; subsequently, p53 mutation/loss-of-function allows the proliferation and persistence of these aneuploid cells. It can be argued that the net result of these two events is tumor progression and poor disease outcomes. Thus, TP53-p21-DREAM exert crucial brakes on G2/M transcription driven by MuvB-FOXM1 and keeps both centrosome amplification and clustering pathways in check.

### 10.5. AR and SPDEF: Vital Restraints on Runaway FOXM1 Expression

While trying to gain insights into connections between the expression of centrosome clustering genes and AR, we found literature reports that AR induces the expression of a SAM-Pointed domain containing ETS transcription Factor (SPDEF). SPDEF was initially recognized as a stimulator of prostate-specific antigen expression [133]. SPDEF expression has been observed to be 10-fold or higher in 24% of primary TNBCs in comparison to normal breast tissue [74]. Among the TNBCs, our analyses found a strong positive correlation between the expression of AR and the expression of SPDEF (Figure 4). SPDEF normally collaborates with the p53-p21-DREAM pathway to attenuate the expression of FOXM1 by disrupting the positive feedback loop that drives FOXM1 expression [129]. AR and p53 thus work in partnership to normally maintain FOXM1 expression in check and in doing so, maintain the expression of centrosome amplification and clustering genes under tight control. However, in the event that AR is underexpressed (e.g., in AR-low TNBCs) or when p53 is mutated, as it is in ~65–80% of TNBCs [134], these regulatory pathways are defunct and FOXM1 expression is prematurely activated and strongly dysregulated.

Figure 6 depicts how and why the centrosome clustering genes KIFC1, AURKB, BIRC5, and CDCA8, which are all primarily G2/M genes and are extra sensitive to DREAM regulation, are overexpressed in TP53-mutant and/or AR-low TNBCs. Normally, the TP53-p21-DREAM and AR-SPDEF pathways ensure the timely expression of G1/S and G2/M genes (especially FOXM1). Loss of TP53 function leads to premature and enhanced expression of G1/S genes, including E2F1, ATAD2 (a cofactor for E2F1), and numerous genes implicated in centrosome amplification. It also drives the premature and enhanced expression of G2/M genes, especially FOXM1. Low activity of the AR-SPDEF pathway (in AR-low TNBCs) further deregulates FOXM1 expression, causing an abnormal build-up of this oncoprotein. Activation of abnormally high levels of FOXM1 at the G2/M boundary leads to overexpression of hundreds of FOXM1 target genes, including genes that drive centrosome amplification and clustering, proliferation, chemoresistance, and resistance to apoptosis, precipitating a poor prognosis. The model sheds light on connections between centrosome amplification and clustering and shows how these pathways are coordinately upregulated with proliferation and survival pathways in TP53-mutant and AR-low TNBCs. Importantly, our model highlights how a nexus of deregulation surrounding FOXM1 drives the tumor biology of AR-low TNBC.

## 11. Full Circle: Perspectives on a Novel Actionable Biomarker for AR-Low TNBC

It is well known that cells with amplified centrosomes survive by upregulating centrosome clustering pathways; however, the mechanisms by which they optimally index clustering pathway activation to levels of excess centrosomes are poorly understood. Thus, the burning question undergirding the arc of this article is regarding the mechanisms that yoke centrosome clustering pathways to centrosome amplification pathways in AR-low TNBC, which is a breast cancer subgroup notorious for its rampant centrosome amplification and aggressive disease course. The compelling rationale behind investigating this question is that the identification of such a coupling mechanism could identify a critical poor prognosis biomarker as well as a potential therapeutic target that could allow us to undermine cancer cells’ ability to thrive by both generating and efficiently clustering excess centrosomes. Blind spots in our understanding of the molecular underpinnings of QNBC tumor biology and the current paucity of robust patient-stratifying biomarkers and precision treatment targets for AR-low TNBCs provide the impetus for this inquiry. Our analyses demonstrate a pan-cancer pattern of upregulation of crucial centrosome clustering proteins, especially CPC components. In breast cancer datasets, we found significant upregulation of both centrosome clustering proteins KIFC1, AURKB, BIRC5, and CDCA8 and the oncogenes FOXM1, ATAD2, and E2F1 in tumor samples compared to normal samples. Both these gene sets were highly overexpressed in AR-low TNBCs, breast tumors of African Americans, and TP53-mutant breast cancers. Overexpression of each of these genes resulted in poorer recurrence-free survival among breast cancer patients. Our analyses also showed that in AR-low TNBCs, these two gene sets are in overdrive and result in the profound dysregulation of cell cycle controls and proliferation, which underlie poor outcomes. Thus, MuvB-FOXM1-driven transcriptional upregulation is a frontrunner for a tumor-specific and evolutionarily advantageous mechanism that co-upregulates centrosome clustering gene expression with high levels of centrosome amplification-driven gene expression in AR-low and p53-mutant TNBC. This FOXM1-mediated transcriptional cascade ultimately leads to an aggressive disease course, poor outcomes, and racial disparities in outcomes.

By transcriptionally coupling the expression of centrosome clustering genes and centrosome amplification genes, FOXM1 tackles the inescapable need of cancer cells to effectively cluster any extra centrosomes present in the cell so that the cell’s survival is not imperiled by multipolar cell division. Transcriptional coupling eliminates the need to actively track levels of centrosome amplification and instead allows a functional balance of amplification drivers and clustering proteins to be maintained by default. It is through the optimally coupled use of these two evolutionarily adaptive capacities—centrosome amplification and centrosome clustering—that cancer cells have the highest dividend and the transcription factor FOXM1 offers AR-low TNBC cells this critical capacity. FOXM1 additionally gives cancer cells with a high level of regulatory flexibility as it interacts with various regulatory proteins and pathways, allowing it to coordinate centrosome numbers and optimal clustering in diverse environments, ultimately leading to disease progression. In a pan-cancer analysis encompassing 18,000 tumors, the expression of FOXM1 and its co-expressed transcriptional network was the strongest prognosticator of poor outcomes [135]. Our work thus illuminates a profound dysregulation of this cell cycle-regulated network and facilitates a deeper understanding of the tumor biology of AR-low TNBC and potentially QNBC while also pointing to possible treatment targets that might specifically benefit this high-need patient subgroup.

It is our view that p53-mutant and AR-low TNBC cells with supernumerary centrosomes are acutely reliant on FOXM1 for survival, which suggests that FOXM1 and centrosome clustering proteins could be considered potential therapeutic targets and actionable Achilles heels/vulnerabilities in these cells. Unlike centrosome declustering-based treatment strategies (such as the use of KIFC1 inhibitors) that cripple centrosome clustering mechanisms but spare pathways that continue to generate excess centrosomes, pharmacological targeting of FOXM1 would simultaneously disable both evils that help cancer cells flourish. Conveniently, investigation into the development of drugs that inhibit FOXM1 is currently underway. Small molecules or compounds have been identified to specifically bind and inhibit FOXM1 by interfering with its nuclear translocation or inducing its degradation in the cytoplasm [136]. Specifically, the FDI-6 compound inhibited FOXM1 with some efficacy in TNBC xenografts [137]. Combining FOXM1 small molecule inhibitors with other therapies, such as mitotic spindle inhibitors, has been suggested to enhance the success of FOXM1 inhibitors [113]. Thus, co-inhibition of FOXM1 and KIFC1 could be an efficacious strategy for AR-low TNBCs.

Hence, we argue that FOXM1 expression, which functionally couples centrosome amplification pathways to centrosome clustering pathways, would also constitute a superior prognostic biomarker than any amplification or clustering protein alone because the expression of fully coupled variables is considered to be multicollinear and can be replaced by the single variable that couples them (without loss of any critical information). FOXM1 and many centrosome clustering proteins are druggable, and oncogenic FOXM1 is a selective target because it is not expressed in most adult tissues. We assert that by deploying inhibitors of FOXM1, we may be able to downregulate the expansive transcriptional network that FOXM1 helms, which might confer significant clinical benefits for AR-low TNBC and/or QNBC patients, improve their disease outcomes, and aid in the amelioration of persistent racial disparities in breast cancer outcomes.

## Data Availability

The original contributions presented in the study are included in the article, further inquiries can be directed to the corresponding author.
